# Risk of human exposure to arsenic and other toxic elements from geophagy: trace element analysis of baked clay using inductively coupled plasma mass spectrometry

**DOI:** 10.1186/1476-069X-9-79

**Published:** 2010-12-23

**Authors:** Shaban W Al-Rmalli, Richard O Jenkins, Michael J Watts, Parvez I Haris

**Affiliations:** 1 Faculty of Health and Life sciences, De Montfort University, The Gateway, Leicester LE1 9BH, UK; 2 British Geological Survey, Keyworth, Nottingham NG12 5GG, UK

## Abstract

****Background**:**

Geophagy or earth-eating is common amongst some Bangladeshi women, especially those who are pregnant, both in Bangladesh and in the United Kingdom. A large proportion of the population in Bangladesh is already exposed to high concentrations of arsenic (As) and other toxic elements from drinking contaminated groundwater. Additional exposure to As and other toxic elements from non-food sources has not been adequately addressed and here we present the first study to monitor As levels in baked clay (known as sikor).

**Methods:**

Sikor samples originating from Bangladesh were digested using a microwave digester and analysed for their As, Pb, Cd, Mn, Fe and Zn levels using ICP-MS. Detailed As speciation analysis was performed using HPLC-ICP-MS.

**Results:**

Of particular concern were the levels of As (3.8-13.1 mg kg^-1^), Cd (0.09-0.4 mg kg^-1^) and Pb (21-26.7 mg kg^-1^) present in the sikor samples and their possible impact on human health. Speciation analysis revealed that sikor samples contained mainly inorganic As. Modest consumption of 50 g of sikor is equivalent to ingesting 370 μg of As and 1235 μg of Pb per day, based on median concentration values. This level of sikor consumption exceeds the permitted maximum tolerable daily intake (PMTDI) of inorganic As by almost 2-fold.

**Conclusion:**

We conclude that sikor can be a significant source of As, Cd and Pb exposure for the Bangladeshi population consuming large quantities of this material. Of particular concern in this regard is geophagy practiced by pregnant women concurrently exposed to As contaminated drinking water. Future studies needs to evaluate the bioavailability of As and other elements from sikor and their impact on human health.

## Background

The deliberate eating of non-food or non-nutritive substances is known as pica [1]. Many different types of pica have been described in the literature such as ingestion of baby powder, charcoal, calcium hydroxide (lime), ash, uncooked starch and ice [2]. Geophagy is the most common type of pica and involves deliberate eating of earth such as clay. It is an ancient practice that is still widespread in many parts of the world such as in Asia, Africa, South America, North America and parts of Europe [3].

The precise reasons underlying the practice of geophagy remains unknown, although some suggest consumption for nutritional [3,4] and medicinal purposes [5]. Geophagy during pregnancy [3] has often been recommended as a means to increase the intake of some essential elements (especially Ca, Mg, Zn, Fe, Cu, Mn, and Se).

In parts of Africa, soil eating is common amongst females, especially children and pregnant women [6-9]. The prevalence of soil eating amongst pregnant women in Kenya, Ghana, Namibia and Tanzania has been reported in the literature [6-9]. Bangladeshi women residing in Bangladesh [10] and also in the UK consume baked clay known as sikor [11,12]. Although the overall prevalence of this habit amongst the Bangladeshi community has not been determined, it is known to be mostly practiced by pregnant women [12]. Recent studies have suggested that geophagy may be associated with an increased risk of developing anemia [9,13].

The practice of geophagy amongst the Bangladeshi and West Bengal (India) population is of particular concern, as people from these regions are already exposed to high levels of As from drinking contaminated groundwater and rice consumption [14]. Despite the high vulnerability of pregnant women and their unborn babies to this practice, the toxic element intake through geophagy and its potential adverse health effects has not been explored. However, exposure of Bangladeshi pregnant women and unborn babies to toxic elements through drinking water, atmospheric pollution and diet has been reported by various workers. For example, Kile et al. [15] measured the maternal and umbilical cord blood levels of arsenic, cadmium, manganese, and lead in rural Bangladesh and reported that exposure to mixtures of these elements is widespread amongst pregnant women.

It has been reported that some Bangladeshi women may consume as much as 50 to 60 g of sikor per day [12]. Although the content of Fe, Mn, Pb and Zn in sikor samples have been reported previously [12], there are no reports of study on the As content of sikor from Bangladesh. Exposure to As from accidental ingestion of soil by children and adults have been studied extensively for various populations [16,17]. However, there are no published reports on geophagy or accidental soil ingestion in Bangladesh or West Bengal (India).

The aim of the present study was to determine the content of As, Cd, Pb, Fe, Mn and Zn in sikor samples. The Provisional Maximum Tolerable Daily Intake (PMTDI) values for As, Cd, Mn and Pb were estimated and potential health risks discussed.

## Methods

### Sample collection

**Figure 1:**
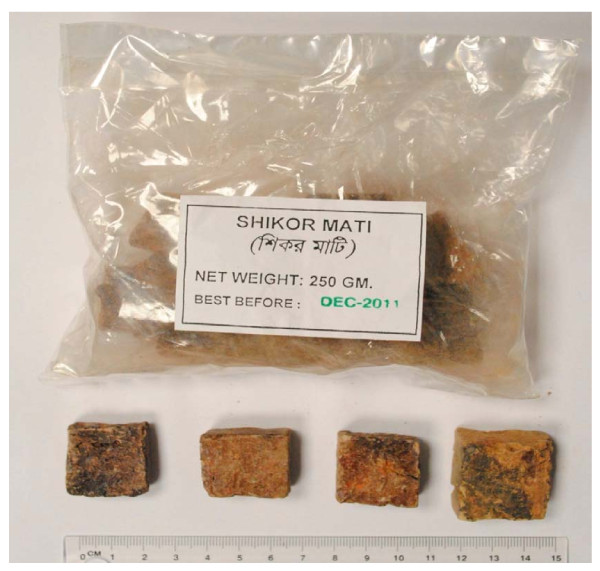
**Typical example of Sikor tablets from Bangladesh purchased from shops in the United Kingdom**. *Shikor Mati *can be translated as Sikor soil.

Eight bags of sikor imported from Bangladesh, openly sold in ethnic Bangladeshi shops in the United Kingdom, were purchased from two cities (Birmingham and Leicester) and one town (Luton) during January to April 2010. These bags contain 250 g of sikor as small tablets (see Figure 1). With the exception of one bag (see Figure 1), there were no labels to indicate the source, composition or expiry date of the product. However, the samples collected from Birmingham, Leicester and Luton appeared to be similar. The weight of the sikor tablets ranged between 12.3 - 18.5 g with a mean of 15 g. According to the shopkeepers, the tablets had been baked in Bangladesh before being imported into the UK.

### Sample preparations

All glassware and plastic were cleaned by soaking in 10% nitric acid (HNO_3_) for at least 12 hours and then rinsed several times with double distilled water. Sikor samples were ground and dried in an oven at 80°C overnight.

### Sample digestion for determination of total As, Cd, Pb, Fe, Mn and Zn content

Sikor samples were digested by microwave assisted digestion in *aqua regia *(1:3 of ultra pure 70% HNO_3 _and pure 37% hydrochloric acid (HCl) were used). A selected weight (0.1 g) of sample was mixed with 5 ml of *aqua regia *overnight and then digested for three hours using a microwave digestion unit at a power of 1000W and a maximum temperature of 170°C (CEM, Microwave digestion MAR *X*press, USA). The solution was made up to 100 ml in volumetric flasks with ultra pure water (Romil-UpS, Ultra Purity water) for analysis.

### Arsenic extraction from Sikor samples

A previously published method was used for the extraction of As from sikor [18]. Briefly, 0.2 g of sikor sample was weighed into a 30 ml bottle and 10 ml of a phosphoric acid (H_3_PO_4_) (1 M)/ascorbic acid (0.5 M) mixture was added. This was mixed for four hours on an orbital shaker at 200 rpm in order to extract the arsenic. Millipore water (20 ml) was then added rapidly to the mixture to avoid conversion from As^III ^to As^V ^[19]. The solution extract was subsequently centrifuged for 15 minutes at 2000 rpm and the supernatant collected. Arsenic levels (total and As species) in all the extracted sample solutions were analysed immediately.

### Instrumentation

#### Analysis of total As, Cd, Pb, Fe, Mn and Zn in sikor samples

Elements in the digested sikor sample solutions were determined by inductively coupled plasma mass spectrometry (ICP-MS), using a Thermo-Fisher Scientific X-SeriesII instrument. For instrument calibration, internal standards were used as follows: Scandium (50 μg L^-1^), Rhodium (10 μg L^-1^) and Iridium (5 μg L^-1^) in the preferred matrix of 2% HNO_3_. Also for calibration, external standards for elements were prepared in the range 0 - 100 μg L^-1 ^(ppb), both an autosampler (Cetac ASX-520) and a concentric glass venture nebuliser (Thermo-Fisher Scientific) were used. The data processing was undertaken using a Plasmalab software (version 2.5.4; Thermo-Fisher Scientific, UK).

#### Arsenic speciation analysis

Detailed elemental speciation analysis was only conducted for As. For this, the total content of As in a solution, extracted from sikor samples, was determined using an Agilent 7500 ICP-MS (Agilent Technologies, UK). This instrument was fitted with a micro flow concentric nebulizer and quartz Scott-type chamber. Helium (4 L min^-1^) was used for collision cell gas and tellurium (50 μg L^-1^) was used as internal standard. Solutions were subjected to arsenic speciation analysis using a HPLC (GP50-2 pump, Dionex, USA) coupled to an ICP-MS (Agilent Technologies, UK). The HPLC conditions used were similar to that previously reported [20]. An anion exchange column (Hamilton PRP-X100, 250 × 4 mm, 10 μm) was used to separate the As species. Ammonium nitrate was used as anion exchange mobile phase at pH 8.65.

#### Methodology for risk estimation

The Joint FAO/WHO Expert Committee on Food Additives (JECFA) [21] method was used to determine the maximum permitted tolerable daily intake (PMTDI) of toxic elements from baked clay. The unit used for this scale is mg of element per day. The average adult body weight of the Bangladeshi women was taken to be 60 kg [21].

#### Quality control and standard reference material

Sample masses were measured to an accuracy of ± 0.1 mg. Trace elements concentrations obtained by ICP-MS technique were evaluated by the use of certified reference materials and found to be in good agreement with the certified values of the reference material. The analytical procedure and the reliability of the digestion process were validated by inclusion of blanks (consisting only of *aqua regia*) with each measurement. Soil reference material (NIST CRM Montana I 2710a) was also included in the same measurement series in order to determine the recovery of the different elements from the sikor samples. The recovery for the soil reference material for the different elements ranged from 75 and 101% (n = 3) of the certified values (Table 1).

**Table 1:** Certified mean values (mg kg-1) for Montana I (SRM 2710a) soil reference material and the mean values we found for selected elements from this sample following aqua regia digestion.

Element	As	Cd	Fe	Mn	Pb	Zn
Certified value	1540 ± 100	12.3 ± 0.3	43200 ± 800	2140 ± 60	5520 ± 30	4180 ± 150

Found value	1430 ± 90	11.3 ± 0.7	34500 ± 320	1610 ± 70	5580 ± 240	3760 ± 80

% Recovery	93	90	80	75	101	80

Detection limit^a^	0.1	0.002	3.9	0.1	0.3	1.3

^a ^Detection limits were calculated as 3 × standard deviation (3 × Std. Dev.) of blank values.

For quality control of As speciation, soil reference material (BCSS-1) was used. The content of total As and As species for this material is known. Both As^III ^and As^V ^were spiked to the soil reference material (BCSS-1) before extraction. The As species were subsequently extracted using the same method that was used for extraction of As from sikor samples (see above). The spike recoveries were up to 100% for both As species, with linearity R^2 ^= 1. The mean recovery of total As extracted from the soil reference material (BCSS-1) was 75 ± 5%, whereas the mean recovery of As species was 76 ± 6%. Very low detection limits were obtained for As species (less than 0.1 mg kg^-1^).

## Results

Typical sikor samples originating from Bangladesh and sold in ethnic shops in the UK are shown in Figure 1. Concentrations of elements in sikor purchased from shops in Leicester, Birmingham and Luton (UK) are given in Table 2, for three toxic elements (As, Pb, Cd) and for three relatively non-toxic essential elements (Fe, Mn, Zn). Regardless of whether mean, median or maximum concentration was applied as a criterion, the concentrations of the six elements were found according to the following order: Fe>Zn>Mn>Pb>As>Cd.

**Table 2:** Concentrations (mg kg-1 unless otherwise indicated) of As, Cd, Fe, Mn, Pb and Zn in Sikor samples.

Element	Mean	SD	Median	Range
As	7.8	2.5	7.4	3.80-13.1

Cd	0.32	0.1	0.34	0.09-0.4

Fe (g kg^-1^)	35.4	4	33.8	29.5-42.9

Mn	57.2	3.2	32.2	25.8-80

Pb	23.2	0.9	24.7	21-26.2

Zn	69.6	15	67.5	51.4-100

Number of samples (n) = 10. All the data presented were obtained for duplicate samples. SD: standard deviation.

Speciation of As from sikor samples by HPLC-ICP-MS showed that the main species was inorganic arsenic (As^V^) at up to 100% of the total extractable As (Table 3). The recoveries for As extraction from sikor samples ranged from 31 to 37%; it is not uncommon for low recoveries in soil samples [19,22,23]. In our study, H_3_PO_4 _(1 M) and four hours contact time was employed, which is widely used for soil and clay extraction [see for example ref. [24]]. Similar results (48% recovery) were obtained in a previous study [24] for As speciation in soil where H_3_PO_4 _(1.6 M) and six hours contact time was used for extraction. However, other studies [20,25] have found that percentage extraction can reach up to 97% using H_3_PO_4_. The low recovery in the present study could be due to the nature of the material such as the high Fe content. It is possible that Fe binds As strongly, preventing its release under the conditions used for the extraction.

**Table 3:** Determination of Arsenic in sikor samples.

Sample	Total As in sikor material (mg kg^-1^)		Extracted As from sikor (mg kg^-1^)		Extraction Efficiency^a ^(%)		As Species (mg kg^-1^)		Column Recovery^b ^(%)	
	
	Mean	SD	Mean	SD	Mean	SD	As^III^	As^V^	Mean	SD
S1	7.2	0.3	2.31	0.02	32	0.2	< LOD	1.91	83	6

S2	6.7	0.5	2.07	0.12	31	1.8	< LOD	1.57	76	1

S3	6.3	0.4	2.07	0.05	33	0.8	< LOD	1.12	54	10

S4	7.4	0.6	2.71	0.06	37	0.7	< LOD	1.21	45	5

S5	5.5	0.3	1.87	0.01	34	1.2	< LOD	1.03	55	10

S6	7.1	0.5	2.18	0.07	31	0.9	< LOD	1.64	75	4

S7	8.1	0.7	2.78	0.04	34	0.9	< LOD	1.87	67	5

S8	7.4	0.4	2.28	0.03	31	0.8	< LOD	1.88	82	3

^a ^Extraction efficiency based on percentage of As which is extracted from sikor samples relative to the total As present in the material. ^b ^Column recovery refers to As species recovered from the column as a percentage of the total As in the extract. < LOD = not detected or low limit of detection. SD: standard deviation. All the data presented were obtained for duplicate samples.

Abrahams et al. [12] reported that sikor, equivalent to 3 - 4 tablets of their Birmingham sample (i.e. ca. 48.6 - 64.8 g), can be consumed per day by pregnant Bangladeshi women. Similar levels of soil ingestion were reported for pregnant Kenyan women, with a median daily intake of 41.5 g from soil sample [26]. In light of these studies, we have selected a modest 50 g of sikor consumption per day for our estimation of the PMTDI of different elements. Table 4 presents the estimation of the maximum daily intake (PMTDI) of As, Cd, Mn and Pb through ingestion of 50 g of sikor; median As content was 370 μg in this quantity of sikor. These data shows that ingestion of 50 g of sikor can exceed the PMTDI for As by almost 2-fold and for Pb by almost 5-fold. For both Cd and Mn, however, consumption of the same amount of sikor contributed only a proportion of PMTDI: 28.3 and 13.4% of PMTDI for Cd and Mn, respectively.

**Table 4:** Percentage of permitted maximum tolerable daily intake (PMTDI) for different elements (median values were used) associated with consumption of sikor.

Element	PMTDI (μg kg^-1^-bw day^-1^)^a^	PMTDI (μg day^-1^)^a^	Percentage of PMTDI from 50 g of sikor
As	2.1	126	293

Cd	1.0	60	28.3

Mn	200	12000	13.4

Pb	3.5	210	588

^a ^The numerical values shown are the tolerable daily intake for a 60 kg person derived from PMTDIs recommended by the Joint FAO/WHO Expert Committee on Food Additives (JECFA)^14^

Abrahams et al. [12] determined some elements in sikor from Bangladesh including Fe, Mn, Pb and Zn, but not As and Cd. The study reported mean concentrations (mg kg^-1^) of 58800 (Fe), 69 (Mn), 80 (Pb) and 38 (Zn) in sikor from Birmingham. Whilst the reported values for Pb are higher compared with our results (21 - 26.7 mg kg^-1^), this could be at least partially explained by differences in digestion procedures; recovery for Pb in the Abraham's study was 264% compared to 101% recovery in the present study.

## Discussion

ICP-MS analysis of sikor samples sold in United Kingdom and in Bangladesh for human consumption reveals the presence of a mixture of toxic elements (As, Cd and Pb). The As content in clay from Bangladesh has been reported to vary from 3.53 to 6.64 mg kg^-1 ^[27]. In another study, concentrations of As, Pb and Zn in clay sediments from Bangladesh were found to range between 4 - 18, 13 - 32 and 35 - 111 mg kg^-1 ^respectively [28]. However, in the Bengal Delta in India, the As content in clay has been found to range between 4 - 10 mg kg^-1 ^[29]. These reported levels are comparable with the levels we have found in our analysis of sikor samples. It appears that the As content of sikor samples are what is commonly found in soil and is unlikely to originate from a contaminated land.

Our speciation analysis revealed that sikor consists primarily of inorganic As. The relatively modest level of daily sikor consumption alone (excluding As intake from water and foods) exceeds the PMTDI for inorganic As by almost 1.9-fold. This finding has relevance not only to the As exposed population in Bangladesh whose total daily As intake from all sources can exceed PMTDI by 4-fold, but also to sikor consumers in the UK who do not normally exceed PMTDI for inorganic As. Pregnant women are of particular concern in this context since As, Pb [30], Cd [31], and Mn [32,33] can transfer from the mother to the foetus, placing the health of the unborn baby at risk. Due to the complex mineral composition of sikor, it is likely that not all the arsenic content would be bioaccessible. Bioaccessibility can be defined as the fraction of the total amount of a substance released from the soil during digestion. A previous *in vitro *study has shown that the bioaccessibilities of As and Cd from soils were higher than those for Fe, Mn and Pb; the mean percentage range was 9.7 - 28.7 and 12.9 - 27.2% for As and Cd, respectively [34]. Further research is required to determine the bioaccessibility of As from sikor.

The Pb content determined in our study is within the range previously reported for Bangladeshi clay by other workers 13-32 mg kg^-1^[28]; both studies reveal high Pb content in sikor samples, which is a health concern for consumers of this material. Exposure to elevated levels of Pb has been associated with various diseases including lung cancer [35], immnuotoxicity [36] and neurotoxicity [37]. Due to increased industrialization and use of Pb based fuels and chemicals, exposure to Pb in Bangladesh is becoming a significant problem and elevated blood Pb levels were detected in primary school children in Dhaka (Bangladesh) [38]. Furthermore, Kile et al. [15] also reported high levels of Pb in maternal and umbilical cord blood from women in rural Bangladesh. From the current study, the PMTDI of Pb can be exceeded by 6-fold through ingestion of 50 g of sikor per day. Such a high level of exposure could present health problems to all women, and pregnant women are at risk of harming their unborn child.

Recently, Kippler et al. [39] reported the effect of Cd on human health in Bangladesh. They found higher Cd levels in breast milk in Bangladeshi women. The mean concentration of Cd in breast milk in Bangladeshi women was found to be 0.14 μg L^-1 ^which was higher than other countries excluding Japan and India [39]. Similarly, Kile et al. [15] also found high levels of Cd in maternal and umbilical cord blood from rural Bangladeshi women. The quantity of Cd in 50 g of the sikor samples we analysed represents 28.3% of PMTDI. If Cd intake through consumption of leafy vegetables, which also contain high levels of Cd, is taken into account there is potential for exceeding the PMTDI for this element. For women living in rural Bangladesh the daily calorie intake is dominated by vegetables and rice, since animal products - which have lower Cd content- are often too expensive [40]. Moderate intake of fish is the main animal based product in the diet of many Bangladeshis who cannot readily afford to purchase poultry and meat [40]. Thus a diet already rich in Cd, due to high rice and vegetable intake, is worsened by the consumption of sikor. In addition, we have recently found that betel quid chewing can be an additional source of Cd exposure in Bangladeshi women (article submitted). In light of our studies, it is plausible to state that some of the excess Cd detected in the breast milk of Bangladeshi women [39] may come from the consumption of non-food materials such as sikor.

Very little data is available on the prevalence of sikor consumption amongst Bangladeshi women in Bangladesh and in the UK. Due to the stigma associated with the practice, many women are ashamed to admit that they consume sikor. However, the commercial availability of sikor in both the UK and Bangladesh clearly indicates there is a demand for the substance in both countries, although it is not possible to translate this information into number of consumers. Consumption of sikor in relatively large quantities by pregnant women has recently been highlighted in Bangladesh [10]. A gynaecologist working in a Bangladeshi hospital, interviewed by the press, reported that many of her pregnant patients consume sikor in large quantities [10]. In parts of Africa earth-eating is openly practiced and is an acceptable social habit and, consequently, the scientific research on earth-eating in African women is much more extensive compared to Bangladesh and India. Its prevalence among pregnant women ranged from 65% in Kenya, 46% in Ghana, 42% in Namibia, to 29% in Tanzania [6-9].

Previously it has been reported that the major minerals in sikor from Bangladesh are kaolin, illite and quartz [12]. It was found to be very low in organic carbon (0.8%) and is essentially salt free. Sikor is considered to be a source of nutrients due to its high concentrations of essential elements. Our study reveals that the level of the essential elements (Fe, Zn and Mn) can either contribute towards the recommended daily intake of these elements or can result in an excess intake for those with already high intake of Fe, Mn and Zn. However, at the same time our study shows that it may also be harmful to human health due to the presence of the toxic elements As, Pb and Cd. Therefore, depending on the nutrient status of the consumer, ill health can result from either exposure to toxic elements and/or overexposure to essential elements from ingestion of sikor. How sikor consumption influences uptake of different elements (essential or toxic) *in vivo *needs to be determined in order to evaluate its impact on human nutrition and health.

We estimate that consumption of just 50 g of Bangladeshi sikor per day can result in the PMTDI for As and Pb being exceeded by 3- and 6-folds, respectively. Sikor consumption should therefore be considered as potentially harmful to consumers in the UK who can purchase this material from ethnic shops in cities with a sizeable Bangladeshi community. In the UK, the Bangladeshi community has a diet which is very similar to their country of origin and consume foods imported from Bangladesh and are at greater risk of exposure to As especially due to their high intake of rice [41]. The risks associated with sikor consumption are likely to be greatly exacerbated for consumers in Bangladesh, especially those who are already exceeding their daily intake of As from consumption of contaminated water and foods such as rice.

## Conclusions

This study reveals for the first time that baked clay, consumed by Bangladeshi women, can be a source of arsenic exposure that has not been previously considered in risk assessment studies. The simultaneous presence of Cd, Pb and Mn in sikor is also a cause for health concern. Since sikor is consumed more often, and in higher quantities during pregnancy, by Bangladeshi women both in Bangladesh and in the UK, the potential adverse health and developmental effects to the unborn baby is of particular concern. As, Cd and Pb can generate reactive oxygen species in biological systems which can adversely affect the cells and organs in the mother and the foetus. The authors recommend that those responsible for public health act to create awareness about the potential dangers of consuming baked clay in populations where this practice is prevalent.

## Abbreviations

CRM: certified reference material; ICP-MS: inductively coupled plasma - mass spectrometry; PMTDI: permitted maximum tolerable daily intake.

## Competing interests

The authors declare that they have no competing interests.

## Authors' contributions

The study was designed by SA and PH. The total contents of As, Cd, Pb, Fe, Mn and Zn analysis were undertaken by SA. As speciation analysis was conducted by SA and MW. The first draft of the manuscript was undertaken by SA and comments and changes were made by PH, RJ and MW. All authors have approved the final manuscript.
